# Susceptibility results discrepancy analysis between NHSN antimicrobial resistance (AR) Option and NEDSS Base System in Tennessee, July 2020–December 2021

**DOI:** 10.1017/ash.2023.377

**Published:** 2023-09-29

**Authors:** Carol Davis, Youssoufou Ouedraogo, Christopher Evans, Christopher Wilson

## Abstract

**Background:** The NHSN Antimicrobial Resistance (AR) Option is an important avenue for acute-care hospitals to electronically report facilitywide antibiogram data. The NEDSS Base System (NBS) is the statewide surveillance system for mandatory reporting of all carbapenem-resistant Enterobacteriaceae (CRE) cases. The state health department (SHD) validated CRE case data reported through the AR Option to assess completeness and accuracy. **Methods:** NHSN AR Option data from July 2020–December 2021 for 24 facilities were validated by comparing reported CRE and susceptibility results to CRE isolates reported via the NBS. Isolates were matched based on specimen date, sex, birth month and day, pathogen, and specimen source. NHSN susceptibility results were dichotomized as “not resistant” and “resistant” to match the NBS results. Susceptibility discordance (differing proportions of resistant isolates) of matched pairs were evaluated using the McNemar exact test in SAS version 9.4 software. **Results:** The SHD identified 270 CRE cases from the NHSN and 1,254 unique CRE isolates from the NBS. Of the NHSN events, 72 (26.67%) were matched to the NBS. Among matched isolates, discordance was significant for doripenem (0 resistant isolates in the NHSN vs 13 in the NBS; *P* < .001) and imipenem (5 resistant isolates in the NHSN vs 23 in the NBS; *P* < .0001). Discordance was not significant for ertapenem nor meropenem. Sensitivity analyses maximized the match rate at 30.74% (83 matches) when NBS isolates from unknown sources were included and matching factors were specimen date and date of birth ± 1 day, and pathogen. Among all 6,325 CRE isolates in NBS, 290 (4.58%) did not have a specimen source provided. Of all 47,348 NHSN events, 7,624 (16.10%) had impossible patient birthdays. **Conclusions:** Many NHSN isolates could not be matched to NBS due to either isolates being missing from NBS or to data differences across the systems. This mismatch highlights the need for data validation and standardization at the point of entry for both systems. Discordant susceptibility outcomes raise concerns about using the NHSN as a method for facility and regional antibiogram data.

**Disclosures:** None

